# An Overview of Biomarkers for the Diagnosis of Hepatocellular Carcinoma

**DOI:** 10.5812/hepatmon.6122

**Published:** 2012-10-20

**Authors:** Mona Abu El Makarem

**Affiliations:** 1 Internal Medicine Department, Minia University, Minia, Egypt

**Keywords:** Carcinoma, Hepatocellular, Diagnosis, Bioligical Markers

## Abstract

**Context:**

Primary liver cancer is one of the most common and deadly malignant neoplasms worldwide. The incidence and mortality rates for hepatocellular carcinoma (HCC) are virtually identical, reflecting the poor overall survival of patients with this kind of tumor. Effective therapies mostly achieved if the HCC diagnosis is made at early stages of the tumor. Surveillance tests include serologic and radiologic examinations.

**Evidence Acquisition:**

In this review, an overview of biomarkers for the diagnosis of HCC and future challenges in this popular field has been presented.

**Results:**

Serum tumor markers, such as alpha-fetoprotein (AFP) and des-gammacarboxy prothrombin (DCP) are commonly used for the surveillance, but their roles have been intensely debated despite the existence of sensitive radiologic tests. Most HCC-related cancer biomarkers are involved in chronic inflammation and cancer. These biomarkers, according to their biologic characteristics are primarily divided into three groups including onco-foetal protein, stress protein, and post-translational modification.

**Conclusions:**

Because of the limitations of traditional HCC biomarkers, exploration for novel biomarkers for the diagnosis of HCC is an evolving process.

## 1. Context

Primary liver cancer is one of the most common and deadly malignant neoplasms worldwide. Globally, it is the fifth most common cancer and the third most common cause of deaths by cancer, behind lung and stomach cancers (1-3) Hepatocellular carcinoma (HCC) accounts for most primary cancers of the liver. HCC is the most common malignant neoplasm in several regions of Africa and Asia. At least 300,000 of the 600,000 deaths from HCC worldwide occur in China, and most of other 300,000 deaths occur in resource-challenged countries in sub-Saharan Africa. This dramatic rise in the prevalence of HCC is presumably associated with chronic hepatitis B and C (2, 3). The emergence of the hepatitis C virus (HCV) in developed countries accounts for approximately half of this increase in HCC (1-4). In Japan, the United States, Latin America, and Europe, hepatitis C is the major cause of HCC. The incidence of HCC is 2% to 8% per year in patients with chronic hepatitis C and established cirrhosis. In Japan, mortality rate owing to HCC has been more than tripled since the mid-1970s; HCV infection is responsible for 75% to 80% of the cases. In Asia, Africa, and some eastern European countries, chronic hepatitis B is the primary cause of HCC, far outweighing the impact of chronic hepatitis C. There are 300 million people infected with HBV, which 120 million are Chinese. In China and Africa, hepatitis B is the major cause of HCC; approximately 75% of the HCC patients have hepatitis B. The etiology of HCC in 15% to 50% of new cases still has remained unclear, which suggests that other risk factors likely account for the increase (5). In Egypt, the incidence of HCC has been nearly doubled over the last decade (6, 7), and Egypt has simultaneously been plagued with the highest prevalence of HCV in the world, ranging from 6% to 28% (8, 9). The prevalence of serological markers of HCV infection in patients with HCC is nearly 80% (4). Of all the cancer sites, HCC represents the leading cause of death (7). Interestingly, the incidence of HCC in developed countries including Japan, Australia, European countries, Canada, and the United States has been increased over the last 20 years (10, 11) . In the United States alone, the annual incidence of HCC has increased by approximately 80% over the last two decades (2). The incidence and mortality rates for HCC are virtually identical, reflecting the poor overall survival rates for patients with this kind of tumor. Most therapies are only effective if HCC is diagnosed at early stages(12). HCC presents two relevant concerns: i) the presence of a cirrhotic background that severely affects both the quality of life and the survival of the patients, and ii) the pleiotropic pathogenesis possessing a common background: chronic inflammation and oxidative stress.

## 2. Evidence Acquisition

Surveillance tests include serologic and radiologic examinations. Most HCC-related cancer biomarkers are due to chronic inflammation and cancer. These biomarkers according to their biologic characteristics are divided into three groups, comprises the onco-foetal protein, stress protein, and post-translational modification groups. The efficacy of serum biomarkers based evaluation is still limited. Serum tumor markers, such as alpha-fetoprotein (AFP) and des-gammacarboxy prothrombin (DCP), are commonly used for the surveillance, but their roles are being intensely debated despite existence of sensitive radiologic tests. Even though, the presence of these markers sometimes overlaps during the diagnosis of HCC, a combination assay of at least two or three markers is recommended for a more sensitive and specific diagnosis of HCC. However, these traditional biomarkers do not reflect the biological features of the tumor or provide information about HCC behavior; thus, they do not allow the physician to accurately predict the outcomes of HCC patients (13). In the emerging era of new molecular-targeted therapy for HCC, the evaluation of these novel agents will also require novel tools. Well-established concepts in oncology may be no longer valid, which indicates that there is much room for improvements in both the efficacy of the traditional biomarkers as well as other serological markers. Multiple efforts are now being directed towards the discovery of novel HCC biomarkers. Recent advances in genomics and proteomics could provide novel tools to improve the diagnostic and prognostic prediction of HCC. Biomarkers derived from microarray expression-profiling data can be subject to high false positive rates because of the multiple hypotheses inherently being tested when working with large numbers of genes and gene combinations. A predictive biomarker signature or gene set determined from a given set of samples (the training set) must be validated with data from independent samples (the test/validation set) (14). Meeting this goal can be challenging because independent data sets, especially those from clinical samples treated in a similar way, are scarce or it requires a significant time investment to accumulate. One workaround to this limitation is the formulation and elucidation of novel serum HCC biomarkers with high diagnostic accuracy. In this review, an overview of biomarkers for the diagnosis of HCC has been made.

## 3. Result

### 3.1. Hepatocellular Carcinoma Specific Biomarkers

#### 3.1.1. Alpha-Fetoprotein

Alpha-fetoprotein (AFP) is the most common and classical tumor marker used for HCC evaluation. Bergstrand and Czar (15) discovered AFP in 1956 using paper for its electrophoretic separation from human fetoprotein in serum. Tatarinov, in 1964, and Abelev (16), in 1968, presented the first reports on the usage of AFP as a diagnostic marker for HCC. AFP is a glycoprotein with a molecular weight of approximately 70 kDa, which is synthesized in the endodermal cells of the yolk sac during early fetal development and subsequently in embryonic hepatocytes (17). It reaches a maximum serum concentration of 3 g/L in weeks 12-16 of fetal life and during the next 18 month to this time. The AFP values subsequently decrease (18). The human AFP gene has been linked to chromosome 4 (4q11–q13) which is part of the albuminoid gene superfamily. Plus AFP, this gene encodes several proteins including albumin and vitamin D-binding proteins (19). AFP presumably functions as a transport molecule for several ligands, such as bilirubin, fatty acids, retinoid, steroids, heavy metals, dyes, flavonoids, phytoestrogens, dioxin, and various drugs (20). AFP is thought to exhibit immunosuppressive activity, it also plays a role in regulation of cell proliferation (21). Its synthesis in adults is repressed. Pathological elevation is detected during hepatocyte regeneration and hepatocarcinogenesis. Despite the existing uncertainty concerning its biologic role, an increase in the serum concentration of AFP is primarily used as a tumor marker for HCC evaluation.

Numerous data have proved that significantly higher AFP serum levels associated with various liver diseases, such as viral hepatitis, liver cirrhosis, and liver tumors (primarily HCC and hepatoblastoma, but also metastasis in 5%-10% cases), and other neoplasms, which are the most prevalent cancers of the digestive tract (pancreas ~24%, stomach ~15%, large intestine ~3%, and gallbladder). The low specificity of AFP as a diagnostic tool for HCC is a clinical problem. In contrast, fucosylated AFP (AFP-L3) is a more specific marker for HCC when compared with AFP alone (22). Recently, Marrero et al. reported that a new cut-off for AFP gave a higher sensitivity than either AFP-L3 or Des-gamma-carboxy prothrombin (DCP) (23). Paradoxically, these data suggest the low significance of AFP-L3 and DCP as markers for the early detection of HCC. Additional research is required to assess the value of these markers in clinical trials. In addition, the positive predictive values (PPV) for AFP are significantly lower among patients with an HCC viral etiology than a non-viral etiology (PPV: 70% vs. 94%, respectively, P < 0.05) (24). It has been confirmed in numerous investigations that AFP serum concentration increase in parallel with HCC tumor size. AFP specificity varies from about 76% to 96% and this parentage is improved with the elevation of cut-off value (25, 26) (Table 1).

**Table 1.** Diagnostic Values of Alpha-Fetoprotein as a Hepatocellular Carcinoma Biomarker

	Cut-off value, mg/L	Sensitivity, %	Specificity, %
**Gambarin-Gelwan M. *et al*. (2000) and Kokudo N. *et al.* (2009) (25, 26)**	20	55-60	88-90
**Kokudo N. *et al.* (2009) (26)**	50	47	96.0
**Gambarin-Gelwan M. *et al.* (2000) (25)**	100	31.2	98.8
**Gambarin-Gelwan M. *et al.* (2000) (25)**	200	22.4	99.4
**Gambarin-Gelwan M. *et al.* (2000) (25)**	400	17.1	99.4

#### 3.1.2. DCP (PIVKA-II)

DCP was identified as an HCC biomarker in 1984. DCP an abnormal prothrombin protein induced by antagonist II (PIVKA-II) or the absence of vitamin K. It represents an abnormal product of liver carboxylation during the formation of thrombogen that acts as an autologous mitogen for HCC cell lines (27, 28). Increased levels of DCP are most notably found in advanced cases with portal vein invasion (29). DCP is potentially valuable primarily as a prognostic biomarker, which would be predictive of rapid tumor progression and provide idea about possibility of poor prognosis (30). Besides its utility for HCC screening, serum DCP can also be used as a clinicopathologic or prognostic indicator for HCC patients and potentially to be more helpful than AFP in reflecting the aggressive invasive distinctiveness of HCC. It has been reported that approximately 30% of AFP-negative HCC is DCP-positive. These sub groups of HCC patients who are DCP seropositive and AFP seronegative usually display a higher frequency of HCC possessing a distinct margin, large size nodules of more than 3 cm, few nodules, and moderate to poor differentiation (31). Notably, simultaneous determination of the serum DCP concentration and tissue DCP expression is of synergistic value than assessment of any of these factors alone for predicting the prognosis of HCC patients (32). For a small HCC, measurement of both tumor markers is recommended, since DCP is a more specific marker compared with AFP (33). A high DCP level implies a poor prognosis, and a slight increase in the DCP concentration after therapy could suggest recurrence.

Interestingly, DCP has a biological function in HCC growth. Suzuki et al. reported that DCP acts as a growth factor in both an autocrine and paracrine manner (28).

DCP is a novel type of vascular endothelial growth factor that possesses potent mitogenic and migrative activities (34). DCP stimulates cell proliferation in HCC lines through the activation of Met-Januskinase 1 signal transducer and an activator of the transcription three signaling pathway. Moreover, DCP can induce both cell proliferation and migration of human umbilical vein endothelial cells. Several reasons put DCP forward to be an important tumor marker in the daily clinical practice. Beside that the diagnostic value of DCP as a biomarker is comparable to that of AFP, Grazi et al. (35) proved that AFP and DCP are not correlated, so the combination of those couple of markers can significantly improves HCC detection, with 74.2% sensitivity and 87.2% specificity (Table 2) (36-38). DCP can be analyzed by immunoenzymatic methodology with a higher sensitivity.AFP-L3 and DCP by immunoenzymatic methodology with a higher sensitivity (35).

**Table 2.** Diagnostic Values of the Hepatocellular Carcinoma Serum Biomarkers

	Sensitivity, %	Specificity, %
**AFP-L3 (36, 38)**	61.60	92.00
**DCP (36, 38)**	72.70	90.00
**AFP (36, 38)**	67.70	71.00
**AFP-L3+DCP (36, 38)**	84.80	97.80
**AFP-L3+AFP (36-38)**	73.70	86.60
**DCP+AFP (36, 38)**	84.80	90.20
**AFP-L3+DCP+AFP (36, 38)**	85.90	59.00
**Osteopontin (53)**	95.35	100

#### 3.1.3. Glypican-3 (GPC-3)

Glypican-3 (GPC-3) is an onco-foetal protein and a heparin sulphate proteoglycan that is anchored to the plasma membrane through glycosylphosphatidyinositol (39). In normal situation, GPC-3 is involved in the regulation of cell proliferation and survival during embryonic development and plays a crucial role as a tumor suppressor. GPC-3 show different behaviors among different cancers, while it has been reported to be downregulated in breast cancer, ovarian cancer and lung adenocarcinoma (40), it has been reported to be upregulated in HCC (41). Normally, GPC-3 is absent from the healthy hepatocytes and in patients with a non-malignant hepatocytes. In HCC patients, GPC-3 can be detected in approximately 50% and 33% of HCC patients that are seronegative for both AFP and DCP. The specificity of GPC-3 is 100% (42). Emerging evidence refer to the potential value of the simultaneous determination of GPC-3 and AFP which may impact significantly in increasing the sensitivity of HCC detection without any reduction in the specificity (43).

Another recent study has shown the potential diagnostic value of a couple of novel discovered membranous proteins: Golgi protein 73 (GP73) and mucin 1 (MUC-1). GP73 is a resident Golgi protein, which is upregulated in the hepatocytes of patients with acute hepatitis (44) and cirrhosis (45) and in the sera of patients with HBV- and HCV-related HCC (46). Promising results were reported by Marrero et al. (46) who shows that it exhibit a higher sensitivity (69%) and specificity (75%) in comparison to AFP in discriminating HCC from cirrhotic patients, indicating its superiority in comparison to AF, which has a sensitivity of 30% and a specificity of 96%. MUC-1 is a membrane protein that is expressed in many epithelial cells but it is reported to be overexpressed in patients with breast cancer (47), inflammatory lung diseases (48), and HCC (49, 50). Serious of studies refer to its value in diagnosis of HCC patients. Moriyama et al. (50) reported the expression of MUC-1 in HCC cells and in the serum of patients with HCV-related HCC. Gad et al. (49) reported a specificity of 99% and a sensitivity of 87% for the combination of MUC-1, DCP and AFP in Japanese and Egyptian patients with HCC.

#### 3.1.4. Osteopontin

Osteopontin (OPN) is an integrin-binding glycophosphoprotein that is expressed in several cell types, for instance in particular transformed malignant epithelial cells, and is believed to be involved in many physiological cellular functions such as regulation of migration, invasion, and also metastasis of tumor cells as well as their survival (51). The elevated expression of OPN at mRNA levels has been reported to be associated with the prognosis of HCC patients (52). In a recent study performed by our group (53), the plasma OPN levels were significantly higher in HCV-related HCC patients than in healthy control individuals and also higher than in patients with chronic liver diseases. In contrast to α-fetoprotein, the OPN levels within the HCC group correlated to an advancing degree of the tumor stage indicated by the number of nodules, the size of the tumor nodules, vascular invasion, lymph node metastasis and TNM staging. Additionally, the diagnostic efficacy of OPN was superior to AFP in terms of AUC, sensitivity, specificity, PPV and NPV, whereas the correlation between the OPN and AFP levels was not significant. Ultimately, the diagnostic usage and implication of plasma OPN in HCC needs to be validated in other large multicentre cohort studies.

#### 3.1.5. Other Biomarkers

Multiple other biomarkers are emerging for the diagnosis evaluation of HCC. Squamous cell carcinoma antigen (SCCA) which represents a family of serine proteases of high molecular weight, also known as serpins. Hussein et al. (54), reported the potential value of SCCA in the diagnosis of HCC. The Sensitivity and specificity for SCCA in HCC diagnosis are 77.6% and 84%, respectively. Giannelli et al. suggested that the harmonizing powers which can be gained from (high sensitivity/low specificity) and total AFP (low sensitivity/high specificity) would cause the use of both markers to be of potential added for screening. In fact, this combination leads to a diagnostic accuracy of 90% (55). The concentrations of serum heat shock proteins (HSPs) are a potential tumor marker for HCC evaluation. Given that HSPs are widespread molecules induced in cells, it can be liable easily to exposed to various stress conditions, including carcinogenesis. HSPs have also been identified as a tumor marker for HCC evaluation obtained from proteomic analyses (56). HSP70 could be used as a perceptive marker for the accurate differentiation between early HCC from precancerous lesions or a non-cancerous liver. In daily clinical practice this differentiation is challenge distinction for pathologists owing to the very well differentiated histology with little atypia in early HCC (57). Further studies on HSP70 and HCC at the molecular level are required. miRNA as future biomarkers of hepatocellular carcinoma attaining more attentions nowadays. miRNA expression profiling of HCC was compared in 25 paired HCC patients, using adjacent non-tumorous (NT) tissue samples by miRNA microarray analysis, revealed an increased expression of three miRNAs and decreased expression of four miRNAs in HCC (58) . The significant increased miR-18 and miR-20 abundance in correlation with increase of the poorness of tumor differentiation is suggesting that it may be that altered miRNA expression is contributing to loss of hepatocyte differentiation. Basic evidence from model of HCC revealed 23 unregulated and 4 down regulated miRNAs, notably that miR-122 was the most consistently downregulated miRNA in HCC tissue (59). Our increased understanding of the molecular basis of HCC, and this identification of dysregulated miRNA expression in HCC has led to putting forward of the hypothesis of evaluating the potential value of miRNA target identification as a novel molecular targeted therapy for management of late stages HCC patients. On the other hand the potential value of the assay of altered miRNAs in HCC for predicting the response to HCC therapies deserves further evaluation. Scarce data are available show that by examining miRNA expression profiles in hepatoma cells in comparisonwith human hepatocytes. Twenty six miRNAs including members of the let-7 family were found to be downregulated in hepatoma cells (60). Another clinical finding show that reduced expression of miR-199a-3p in HCC was associated with a significantly decreased time to recurrence in patients who underwent surgical resection (59).

## 4. Conclusion

In conclusion, we have summarized the primary HCC biomarkers (Table 3). While many tumor markers for HCC have been reported in other studies, none of them have been proven to be completely optimal. Despite the fact that the presence of these markers sometimes overlaps during the diagnosis of HCC, a combination assay comprising at least two or three markers is recommended for a more sensitive and specific diagnosis for HCC. AFP is the best clinical HCC marker to date. Although DCP, AFP-L3, and OPN exhibit high specificity regarding the diagnosis of HCC, many clinicians use AFP values to follow-up patients with chronic liver diseases due to its higher sensitivity. The results of conventional tumor markers are negative for approximately 30% to 40% of HCC patients; therefore, searching for novel HCC markers must be continued. SCCA and HSP70 may be considered as key biomarkers for HCC patients when the results for traditional biomarkers are negative.

**Table 3.** Hepatocellular Carcinoma Biomarkers and Their Potential Clinical Use

HCC Marker	Clinical Use
**Alpha-fetoprotein**	Early diagnosis, monitoring, and recurrence
**Des-gamma-carboxy prothrombin (DCP)**	Early diagnosis and prognosis, portal vein invasion and metastasis
**Glypican-3**	Early diagnosis
**Ostepontin**	Early diagnosis, monitoring, and recurrence
**Micro RNAs**	Tumor spread and survival
**Lens culinaris agglutinin reactive AFP (AFP-L3%)**	Early diagnosis and prognosis, vascular invasion

### 4.1. Prospective Aspects

Recent advances in genomics and proteomics could provide a novel tool to improve the diagnostic and prognostic prediction of HCC. Development and progression of HCC is known to be caused by an accumulation of genetic changes that results in the expression of cancer-related genes, such as oncogenes, tumor suppressor genes, and genes involved in many regulatory pathways, including cell cycle control, apoptosis and angiogenesis.

Modern technology enables investigators to measure the expression of thousands of miRNAs simultaneously, which may lead to acquiring some comprehensive information for the diagnosis and therapy of HCC patients. However, it is difficult to detect such molecules in sera of patients with cancer at an early stage, even if high expression of the molecule has been detected in cancer tissues with several arrays. According to novel advances in the management of HCC reported by Llovet et al. (61) in 2008, high accuracy rates have been presented by a 3-gene set, glypican-3, LYVE1 (lymphatic vessel endothelial hyaluronan receptor-1), and survivin. Major limitations for routine use of molecular technology in a clinical setting are currently the cost and access to these technologies. Hopefully, the costs will soon decrease, and this technology will become increasingly more popular and automated. Exploration for novel biomarkers for the diagnosis of HCC is an evolving process.

## References

[1] Gomaa AI, Khan SA, Toledano MB, Waked I, Taylor-Robinson SD (2008). Hepatocellular carcinoma: epidemiology, risk factors and pathogenesis.. World J Gastroenterol.

[2] World Health Organization. (2010). Mortality database..

[3] El-Serag HB, Rudolph KL (2007). Hepatocellular carcinoma: epidemiology and molecular carcinogenesis.. Gastroenterology.

[4] Lehman EM, Wilson ML (2009). Epidemiology of hepatitis viruses among hepatocellular carcinoma cases and healthy people in Egypt: a systematic review and meta-analysis.. Int J Cancer.

[5] Bugianesi E (2007). Non-alcoholic steatohepatitis and cancer.. Clin Liver Dis.

[6] Freedman LS, Edwards BK, Ries LAG (2006). Cancer incidence in four member countries (Cyprus, Egypt, Israel, and Jordan) of the middle east cancer consortium (MECC) compared with US SEER.

[7] (2010). National Cancer Registry of Egypt. Magnitude of hepatocellular carcinoma in Egypt.. http://www.nci.cu.edu.eg/.

[8] (2007). Egyptian Ministry of Health. Egyptian Ministry of Health Annual Report.. http://www.mohp.gov.eg/Main.asp.

[9] Khattab MA, Eslam M, Sharwae MA, Hamdy L (2010). Seroprevalence of hepatitis C and B among blood donors in Egypt: Minya Governorate, 2000-2008.. Am J Infect Control.

[10] Bosch FX, Ribes J, Diaz M, Cleries R (2004). Primary liver cancer: worldwide incidence and trends.. Gastroenterology.

[11] Altekruse SF, McGlynn KA, Reichman ME (2009). Hepatocellular carcinoma incidence, mortality, and survival trends in the United States from 1975 to 2005.. J Clin Oncol.

[12] Bruix J, Sherman M (2011). Management of hepatocellular carcinoma: an update.. Hepatology.

[13] Mann CD, Neal CP, Garcea G, Manson MM, Dennison AR, Berry DP (2007). Prognostic molecular markers in hepatocellular carcinoma: a systematic review.. Eur J Cancer.

[14] Ivanovska I, Zhang C, Liu AM, Wong KF, Lee NP, Lewis P (2011). Gene signatures derived from a c-MET-driven liver cancer mouse model predict survival of patients with hepatocellular carcinoma.. PLoS One.

[15] Bergstrand CG, Czar B (1956). Demonstration of a new protein fraction in serum from the human fetus.. Scand J Clin Lab Invest.

[16] Abelev GI (1968). Production of embryonal serum alpha-globulin by hepatomas: review of experimental and clinical data.. Cancer Res.

[17] Yamashita K, Taketa K, Nishi S, Fukushima K, Ohkura T (1993). Sugar chains of human cord serum alpha-fetoprotein: characteristics of N-linked sugar chains of glycoproteins produced in human liver and hepatocellular carcinomas.. Cancer Res.

[18] Debruyne EN, Delanghe JR (2008). Diagnosing and monitoring hepatocellular carcinoma with alpha-fetoprotein: new aspects and applications.. Clin Chim Acta.

[19] Koteish A, Thuluvath PJ (2002). Screening for hepatocellular carcinoma.. J Vasc Interv Radiol.

[20] Mizejewski GJ (2001). Alpha-fetoprotein structure and function: relevance to isoforms, epitopes, and conformational variants.. Exp Biol Med (Maywood)..

[21] O'Neill G, Tsega E, Gold P, Murgita RA (1982). Regulation of human lymphocyte activation by alpha-fetoprotein. Evidence for selective suppression of Ia-associated T-cell proliferation in vitro.. Oncodev Biol Med.

[22] Li D, Mallory T, Satomura S (2001). AFP-L3: a new generation of tumor marker for hepatocellular carcinoma.. Clin Chim Acta.

[23] Marrero JA, Feng Z, Wang Y, Nguyen MH, Befeler AS, Roberts LR (2009). Alpha-fetoprotein, des-gamma carboxyprothrombin, and lectin-bound alpha-fetoprotein in early hepatocellular carcinoma.. Gastroenterology.

[24] Soresi M, Magliarisi C, Campagna P, Leto G, Bonfissuto G, Riili A (2003). Usefulness of alpha-fetoprotein in the diagnosis of hepatocellular carcinoma.. Anticancer Res.

[25] Gambarin-Gelwan M, Wolf DC, Shapiro R, Schwartz ME, Min AD (2000). Sensitivity of commonly available screening tests in detecting hepatocellular carcinoma in cirrhotic patients undergoing liver transplantation.. Am J Gastroenterol.

[26] Kokudo N, Makuuchi M (2009). Evidence-based clinical practice guidelines for hepatocellular carcinoma in Japan: the J-HCC guidelines.. J Gastroenterol.

[27] Ikoma J, Kaito M, Ishihara T, Nakagawa N, Kamei A, Fujita N (2002). Early diagnosis of hepatocellular carcinoma using a sensitive assay for serum des-gamma-carboxy prothrombin: a prospective study.. Hepatogastroenterology.

[28] Suzuki M, Shiraha H, Fujikawa T, Takaoka N, Ueda N, Nakanishi Y (2005). Des-gamma-carboxy prothrombin is a potential autologous growth factor for hepatocellular carcinoma.. J Biol Chem.

[29] Hagiwara S, Kudo M, Kawasaki T, Nagashima M, Minami Y, Chung H (2006). Prognostic factors for portal venous invasion in patients with hepatocellular carcinoma.. J Gastroenterol.

[30] Suehiro T, Sugimachi K, Matsumata T, Itasaka H, Taketomi A, Maeda T (1994). Protein induced by vitamin K absence or antagonist II as a prognostic marker in hepatocellular carcinoma. Comparison with alpha-fetoprotein.. Cancer.

[31] Okuda H, Nakanishi T, Takatsu K, Saito A, Hayashi N, Yamamoto M (2001). Comparison of clinicopathological features of patients with hepatocellular carcinoma seropositive for alpha-fetoprotein alone and those seropositive for des-gamma-carboxy prothrombin alone.. J Gastroenterol Hepatol.

[32] Toyoda H, Kumada T, Kiriyama S, Sone Y, Tanikawa M, Hisanaga Y (2006). Prognostic significance of simultaneous measurement of three tumor markers in patients with hepatocellular carcinoma.. Clin Gastroenterol Hepatol.

[33] Marrero JA, Su GL, Wei W, Emick D, Conjeevaram HS, Fontana RJ (2003). Des-gamma carboxyprothrombin can differentiate hepatocellular carcinoma from nonmalignant chronic liver disease in american patients.. Hepatology.

[34] Fujikawa T, Shiraha H, Ueda N, Takaoka N, Nakanishi Y, Matsuo N (2007). Des-gamma-carboxyl prothrombin-promoted vascular endothelial cell proliferation and migration.. J Biol Chem.

[35] Bertino G, Neri S, Bruno CM, Ardiri AM, Calvagno GS, Malaguarnera M (2011). Diagnostic and prognostic value of alpha-fetoprotein, des-gamma-carboxy prothrombin and squamous cell carcinoma antigen immunoglobulin M complexes in hepatocellular carcinoma.. Minerva Med.

[36] Carr BI, Kanke F, Wise M, Satomura S (2007). Clinical evaluation of lens culinaris agglutinin-reactive alpha-fetoprotein and des-gamma-carboxy prothrombin in histologically proven hepatocellular carcinoma in the United States.. Dig Dis Sci.

[37] Sterling RK, Jeffers L, Gordon F, Sherman M, Venook AP, Reddy KR (2007). Clinical utility of AFP-L3% measurement in North American patients with HCV-related cirrhosis.. Am J Gastroenterol.

[38] Sterling RK, Jeffers L, Gordon F, Venook AP, Reddy KR, Satomura S (2009). Utility of Lens culinaris agglutinin-reactive fraction of alpha-fetoprotein and des-gamma-carboxy prothrombin, alone or in combination, as biomarkers for hepatocellular carcinoma.. Clin Gastroenterol Hepatol.

[39] Filmus J, Selleck SB (2001). Glypicans: proteoglycans with a surprise.. J Clin Invest.

[40] Filmus J, Capurro M (2008). The role of glypican-3 in the regulation of body size and cancer.. Cell Cycle.

[41] Sung YK, Hwang SY, Park MK, Farooq M, Han IS, Bae HI (2003). Glypican-3 is overexpressed in human hepatocellular carcinoma.. Cancer Sci.

[42] Nakatsura T, Yoshitake Y, Senju S, Monji M, Komori H, Motomura Y (2003). Glypican-3, overexpressed specifically in human hepatocellular carcinoma, is a novel tumor marker.. Biochem Biophys Res Commun.

[43] Capurro M, Wanless IR, Sherman M, Deboer G, Shi W, Miyoshi E (2003). Glypican-3: a novel serum and histochemical marker for hepatocellular carcinoma.. Gastroenterology.

[44] Kladney RD, Cui X, Bulla GA, Brunt EM, Fimmel CJ (2002). Expression of GP73, a resident Golgi membrane protein, in viral and nonviral liver disease.. Hepatology.

[45] Iftikhar R, Kladney RD, Havlioglu N, Schmitt-Graff A, Gusmirovic I, Solomon H (2004). Disease- and cell-specific expression of GP73 in human liver disease.. Am J Gastroenterol.

[46] Marrero JA, Romano PR, Nikolaeva O, Steel L, Mehta A, Fimmel CJ (2005). GP73, a resident Golgi glycoprotein, is a novel serum marker for hepatocellular carcinoma.. J Hepatol.

[47] Croce MV, Isla-Larrain MT, Demichelis SO, Gori JR, Price MR, Segal-Eiras A (2003). Tissue and serum MUC1 mucin detection in breast cancer patients.. Breast Cancer Res Treat.

[48] Tokiya R, Hiratsuka J, Yoshida K, Imai S, Kajihara Y, Imajo Y (2004). Evaluation of serum KL-6 as a predictive marker of radiation pneumonitis in patients with breast-conservation therapy.. Int J Clin Oncol.

[49] Gad A, Tanaka E, Matsumoto A, Wahab MA, Serwah Ael H, Attia F (2005). Assessment of KL-6 as a tumor marker in patients with hepatocellular carcinoma.. World J Gastroenterol.

[50] Moriyama M, Matsumura H, Watanabe A, Nakamura H, Arakawa Y, Oshiro S (2004). Detection of serum and intrahepatic KL-6 in anti-HCV positive patients with hepatocellular carcinoma.. Hepatol Res.

[51] Shevde LA, Das S, Clark DW, Samant RS (2010). Osteopontin: an effector and an effect of tumor metastasis.. Curr Mol Med.

[52] Pan HW, Ou YH, Peng SY, Liu SH, Lai PL, Lee PH (2003). Overexpression of osteopontin is associated with intrahepatic metastasis, early recurrence, and poorer prognosis of surgically resected hepatocellular carcinoma.. Cancer.

[53] Abu El Makarem MA, Abdel-Aleem A, Ali A, Saber R, Shatat M, Rahem DA (2011). Diagnostic significance of plasma osteopontin in hepatitis C virus-related hepatocellular carcinoma.. Ann Hepatol.

[54] Hussein MM, Ibrahim AA, Abdella HM, Montasser IF, Hassan MI (2008). Evaluation of serum squamous cell carcinoma antigen as a novel biomarker for diagnosis of hepatocellular carcinoma in Egyptian patients.. Indian J Cancer.

[55] Giannelli G, Antonaci S (2006). New frontiers in biomarkers for hepatocellular carcinoma.. Dig Liver Dis.

[56] Looi KS, Nakayasu ES, Diaz RA, Tan EM, Almeida IC, Zhang JY (2008). Using proteomic approach to identify tumor-associated antigens as markers in hepatocellular carcinoma.. J Proteome Res.

[57] Chuma M, Sakamoto M, Yamazaki K, Ohta T, Ohki M, Asaka M (2003). Expression profiling in multistage hepatocarcinogenesis: identification of HSP70 as a molecular marker of early hepatocellular carcinoma.. Hepatology.

[58] Murakami Y, Yasuda T, Saigo K, Urashima T, Toyoda H, Okanoue T (2006). Comprehensive analysis of microRNA expression patterns in hepatocellular carcinoma and non-tumorous tissues.. Oncogene.

[59] Kutay H, Bai S, Datta J, Motiwala T, Pogribny I, Frankel W (2006). Downregulation of miR-122 in the rodent and human hepatocellular carcinomas.. J Cell Biochem.

[60] Shimizu S, Takehara T, Hikita H, Kodama T, Miyagi T, Hosui A (2010). The let-7 family of microRNAs inhibits Bcl-xL expression and potentiates sorafenib-induced apoptosis in human hepatocellular carcinoma.. J Hepatol.

[61] Llovet JM, Chen Y, Wurmbach E, Roayaie S, Fiel MI, Schwartz M (2006). A molecular signature to discriminate dysplastic nodules from early hepatocellular carcinoma in HCV cirrhosis.. Gastroenterology.

